# The Relationship between Principals’ Instructional Leadership and Teacher Retention in the Undeveloped Regions of Central and Western China: The Chain-Mediating Role of Role Stress and Affective Commitment

**DOI:** 10.3390/bs13090738

**Published:** 2023-09-04

**Authors:** Qifang Zhan, Xiaohan Wang, Huan Song

**Affiliations:** 1 Faculty of Education, Beijing Normal University, Beijing 100875, China; 202011010036@mail.bnu.edu.cn (Q.Z.); donger6695699@126.com (X.W.); 2 Center for Teacher Education Research, Beijing Normal University, Beijing 100875, China; 3 Taihang Branch of Center for Teacher Education Research of Beijing Normal University, Xingtai University, Xingtai 054001, China

**Keywords:** principals’ instructional leadership, teacher retention, role stress, affective commitment, underdeveloped regions of Central and Western China

## Abstract

Enhancing teacher retention in underdeveloped regions of Central and Western China is an important issue for high-quality education development. By investigating 1558 teachers from nine provinces and municipalities in these regions, this study uses quantitative research methods to analyze the relationship between principals’ instructional leadership and teacher retention and introduces a moderated chain-mediated model. The results show that principals’ instructional leadership and teacher retention are at a relatively moderate to high level in these regions. Principals’ instructional leadership not only has direct effect on teacher retention, but also has indirect effects by three paths: through the separate mediating role of role stress, the separate mediating role of affective commitment, and the chain-mediating role of role stress and affective commitment. Teacher gender negatively moderates the relationship between principal’s instructional leadership and teacher role stress. The sample need to be extended to developed areas to form a comparison. Underdeveloped regions of Central and Western China also need to take more action.

## 1. Introduction

In recent years, the development of rural teachers has been a hot topic [1], especially in China’s deeply poverty-stricken areas, where the total number of full-time teachers decreased from 3.23 million in 2012 to 2.81 million in 2017 [2]. The teachers in the underdeveloped regions of Central and Western China are similar to those in rural areas, both of which face numerous difficulties in retention and development. Therefore, in April 2022, the Ministry of Education and eight other departments in China issued the New Era Basic Education Strong Teacher Program [3], which proposes implementing targeted training programs for excellent teachers in these regions. However, policy implementation has not fully addressed the issue of teacher retention. Existing research on teacher retention mainly focuses on its current situation, effects and influencing factors. As to current situation, both policy analysis and empirical research have received attention. Some scholars have noted that although the Chinese government has attached great importance to promoting the development of rural teachers in recent years, rural teachers in China are unable to meet the needs of rural compulsory education development in terms of quantity, quality, and structure [4]. Wang and Li surveyed 10,356 rural teachers in 30 counties in Yunnan Province and found that nearly 80% of rural teachers in the province had a desire to work in cities [5]. As to effects, research generally indicates that teacher mobility and attrition have a destructive impact on education, and is expected to constrain the quality and development of compulsory education in these regions in the long run. As to effects, most research focuses on external influencing factors of teacher retention, such as policy support, and environmental differences between developed and underdeveloped regions. However, the internal factors are overlooked, which is also the main limitation in this research area.

Relying only on external policy support is no longer sufficient to achieve the goal of retaining rural teachers in China. Scholars have argued that the current external-oriented development of rural education has come to an end, and the internal governance of rural schools is expected to become an endogenous driving force for promoting rural education development in the new era [6]. Among the internal governance factors of the school, principals’ instructional leadership is one of the most important factors that scholars are currently focusing on, as it plays a crucial role in promoting overall school improvement, particularly in terms of teacher learning. Hallinger and Liu’s research suggests that urban principals provide stronger learning-centered leadership than rural principals, which is also related to stronger teacher participation in professional learning [7]. In subsequent research, Hallinger and others have confirmed that success in challenging school environments requires leadership that provides inspiration, guidance, support, and program coherence. Through these leadership behaviors, principals establish trust, laying the foundation for transferring ownership of teaching change to teachers. Without these conditions, additional external resource investment may not achieve policy goals. In underdeveloped regions of Central and Western China, principals’ role tend to focus more on political and managerial aspects, while their leadership in promoting teacher learning is often inadequate [8]. Therefore, principals’ instructional leadership has become a focus of attention in the development of these regions in recent years. Nevertheless, few research studies have focused on the effect of principals’ instructional leadership on teacher retention, and how the effect occurs is also explored insufficiently. Among the few research studies, principals’ trust and support for classroom work is supposed to promote teacher retention [9], and the intermediary effect of job satisfaction and job burnout is documented [10]. However, no research has paid attention to how teachers perceive and identity their own roles under principals’ instructional relationship, and how it acts on teacher retention.

In view of the status quo, this study employs a quantitative survey method which is relatively rare in this research area, to explain why and how principal’s instructional leadership can improve teacher retention. The results show that principals’ instructional leadership significantly affects teacher retention directly and indirectly, through teacher role stress and affective commitment as mediating variables, and teachers’ gender as moderating variable, forming a moderated chain mediation model. At the same time, this study has potential impacts on Chinese policy and practice and contributes to the construction of rural teacher teams in these regions in China.

## 2. Review of the Literature

### 2.1. Principals’ Instructional Leadership and Teacher Retention

The principals’ instructional leadership is an important aspect of instructional leadership. Principals’ instructional leadership means that principals participate directly in teaching by formulating teaching curriculum plans and supervising teachers, aiming to promote students’ academic development [11]. Moreover, principals regulate the behavior of teachers and students by managing and formulating relevant system. Teacher retention is defined as individuals’ satisfaction and intention to maintain their organizational identity [12]. Based on this, this study defines principals’ instructional leadership as how principals formulate regulations, manage teachers, guide teaching, assign daily work in the school organization. Then teacher retention is what extent are teachers willing to stay in their original schools in these underdeveloped regions.

Empirical research affirms that talent management has a positive and significant influence on retention intention [13]. Principals’ instructional leadership has significant effects on teacher retention [9], through administrative support [14] and so on. Principals should use instructional leadership to create an appropriate situation to retain and develop teachers [15]. In view of the limitation of existing research, more suitable theories explaining the correlation between principals’ instructional leadership and teacher retention are essential. Role identity theory can serve as a support.

Role identity theory, originating from sociological research on roles, is a synthesis of role theory and identity theory. Role identity theory can be classified as structural role theory and process role theory. Structural role theory advocates the constraining effect of social structure on individual role identity, emphasizing individual reflection, meaning interpretation, self confirmation, and role understanding. Process role theory highlights social interaction [16], exploring role pressure, role conflict, role ambiguity, role overload, role identity crisis, and role identity construction in social interaction. Therefore, teacher retention affected by principals’ instructional leadership can be seen as a kind of teacher role identity generated by interaction with school organization structure. Specifically, principal, as the core leadership of the school, largely shapes the characteristics of the structural context where teachers are situated, including school culture and climate, and teaching organization [17]. Moreover, role identity is not only an identification to real self which is associated with social structure, but also that to ideal self constructed by imagination. The gap between reality and ideal may influence teachers’ role transformation, which often leads to retention or turnover.

Based on supporting theory and previous research, we propose the following:

**Hypothesis 1.**  *Principals’ instructional leadership is expected to be positively related to teacher retention.*

### 2.2. The Mediating Role of Role Stress and Affective Commitment

Principals’ instructional leadership directly affects teacher stress and professional commitment [18], which can also be supported by role identity theory. Role stress, explored in role identity theory as one of the important concepts, is defined as the negative behaviors generated by roles in organization [19]. Structural role theory believes that status and a series of corresponding expectations make up a potential social structure that affects individuals’ role understanding and role identity [20]. Teacher role identity is shaped by the educational management system [21], whose norms are affected by principals’ instructional leadership. Role identity theory also involves individuals’ social identity, suggesting that people recognize their social status by comparing themselves with others. Then, principals’ instructional leadership can increase teachers’ social identity by providing support and recognition, thereby increasing teacher retention. Empirical research has shown that principals’ instructional leadership affects teacher-level variables through the mediating role of school-level variables such as organizational climate, school organizational innovation, and organizational trust [22]. Specifically, role stress can be divided into three levels, namely, role conflict, role ambiguity, and role overload, which have hypothetical connections as the perception of teacher role stress [23]. Studies have shown that teacher stress has a negative impact on teacher retention [24] in rural teachers [25], ad hoc post teachers [26], kindergarten teachers [27,28], etc. Based on this, we define role stress as the negative effects perceived by teachers due to their roles in school organization, including role conflict, role ambiguity, and role overloaded. Then propose the following:

**Hypothesis 2.**  *Principals’ instructional leadership will positively predict teacher retention through the separate mediating role of role stress.*

Affective commitment is one of the dimensions of organizational commitment, which is defined as the relative strength of an individual’s affective orientation toward, identification with and involvement in a particular organization [29]. Both social identity and self identity play a decisive role in developing affective commitment [30]. Affective commitment is also related to the possibility of turnover [31]. Specifically, individuals’ affective attitudes at work can affect their work behavior [32], and negative affective experiences resulting from dissatisfaction with work can make individuals change their work attitudes and re-evaluate their work, leading to decisions about retention [33]. In the attitudinal affective dimension, teacher trust is an important feature of successful leadership [17]. The premise of teacher trust is that the principal’s behavior is moderate, widely supported, and based on collective interests [34]. The findings based on the Organizational Climate Description Questionnaire (OCDQ) developed by Halpin and Croft show that teachers is expected to show division, intolerance, and indifference when principals lack support and exhibit obstructive and controlling behaviors. In contrast, teachers can be confident in school effectiveness and enhance their loyalty to school when principals are confident, optimistic, good at communication, and resourceful, and when they display high levels of leadership [17]. Moreover, the formation of the social part of a teacher’s role identity is mainly based on their exchange with leaders in school organizations. It is social exchange theory that believes the mutual dependence between individuals and organizations improves social exchange relationships [35]. Organizational commitment is positively related to the ideal degree of the work situation [36]. Principals’ instructional leadership can increase teachers’ profits by providing support and rewards, thereby increasing teachers’ affective commitment and retention. Studies have found that teachers’ social exchange relationships play a mediating role between principal transformational leadership and teacher job satisfaction [37], and transformational leadership can reduce teachers’ role stress through social exchange relationships, thereby improving teacher job satisfaction and organizational commitment [38]. Leader-member exchange (LMX) is based on social exchange theory and shows that leaders’ support and trust make subordinates feel warm, and form trust and respect [39]. Furthermore, it is organizational ownership behavior that can present individuals’ organizational behavior in Chinese cultural situations [40]. Role expectation and role support, which shape the process of teacher role identity, are mainly reflected in the role of principals’ supportive leadership. Research also emphasizes that principals’ role expectation and role support act on teachers’ role cognition and role emotion. Principals’ instructional leadership can improve teachers’ affective commitment and retention by increasing their cognition and understanding of their role, which includes organizational justice [41], work satisfaction [42], and perception of organizational support [43]. Empirical research has also shown that affective commitment can be increased by organizational support while simultaneously being eroded by negative exchanges and negative experiences in work [44]. Organizations can improve individuals’ affective commitment and retention intention by focusing on their affective factors and psychological needs, improving attitudes, and communicating. Studies have also found that affective commitment positively predicts teacher retention [45]. Based on this, affective commitment in this study can be defined as the emotional attitude of teachers towards their roles in school organization, as well as the degree of attachment and identity generated from this. Then we propose the following:

**Hypothesis 3.**  *Principals’ instructional leadership will positively predict teacher retention through the separate mediating role of affective commitment.*

Meanwhile, teacher role stress also positively predicts affective commitment. Existing structural equation models have shown that commitment is a mediator variable between role stress and teacher retention [23]. Role stress negatively affects affective commitment, and role conflict and role ambiguity in role stress are also negatively related to affective commitment [46,47]. Therefore, we propose the following:

**Hypothesis 4.**  *Principals’ instructional leadership is expected to positively predict teacher retention through the chain-mediating role of role stress and affective commitment.*

### 2.3. The Moderating Role of Teacher Gender

Gender difference has also received attention in the investigation of teacher role [48]. There is a common trend in existing research that women experience higher levels of occupational stress in terms of gender specific stressors, such as classroom and student behavior under unfavorable conditions, and interference from work and family [49]. Principals’ instructional leadership can also be seen as one of the gender specific stressors. Existing research on female teachers’ stress has mostly focused on the field of higher education. Research has shown that female teachers generally believe that compared to male colleagues, they get less support and respect from leader. Meanwhile, the poorer the principals’ leadership is, the lower resources and support they can receive [50]. The implicit system in gender relations makes female teachers keenly aware of the relationship between discourse system and power, shaping their professional identity, gender identity, and social identity [51]. Based on this, we propose the following:

**Hypothesis 5.**  *Teacher gender plays a negative moderating role between principal’s instructional leadership and teacher role stress.*

**Figure 1:**
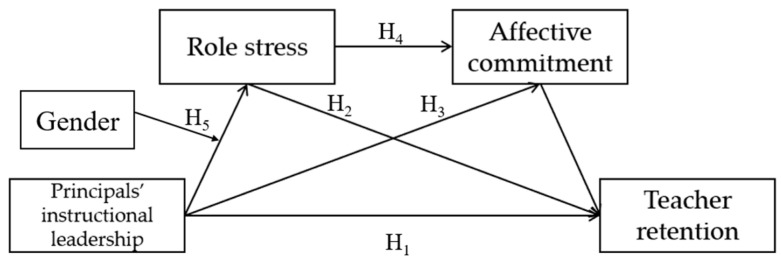
Hypothetical Model.

Thus, we attempt to construct a moderated chain mediation model of principals’ instructional leadership and teacher retention (shown in Figure 1) and test its validity by the regression analysis method.

## 3. Method

### 3.1. Data Sources and Survey Samples

Our study uses a quantitative research paradigm. It targets primary and secondary teachers in the underdeveloped regions in central and western China. Firstly, there are 18 provinces in central and western China, each of which has underdeveloped areas. Then, we selected 9 provinces (Anhui, Shanxi, Hubei, Sichuan, Chongqing, Gansu, Henan, Hunan, and Inner Mongolia) to represent the diversity of overall physical geography and educational scale in these regions. Secondly, this study used online questionnaires to conduct surveys. All teachers included in the sampling range could answer of their own free will. We distributed questionnaires by contacting county-level teaching and research staff, personnel from teacher training institutions, and school principals to forward. The survey period is from March 2021 to May 2021. A total of 2491 questionnaires were collected, and 1558 questionnaires were valid, accounting for a response rate of 62.55%.

Among the teacher samples, the number of female teachers is 1002 (61.3%) while that of male teachers is 556 (35.7%). The teaching experience of teachers is divided into less than 10 years, 10 to 20 years, and more than 20 years (20.0%, 40.6%, and 39.4%, respectively). The number of Han nationality teachers (1532, 98.3%) far exceeds that of minority nationality teachers (26, 1.7%). Teachers in rural and town account for 98.4%, only 1.6% teachers in the urban. As to highest education level, bachelor’s makes up the greatest proportion, at 79.1%, followed by junior college or associate degree (16.8%), while the rest is Master’s or PhD or above (2.1%), Middle school/vocational high school or below (1.2%), primary school or below (0.6%) and others (0.2%). A total of 80.3% teachers major in teacher training, while only 19.7% teachers do not. As to stage, 55.3% teachers work in primary school, followed by 34.9% in junior high school and only 9.9% on senior high school. As to titles, first level teachers constitute the largest proportion, at 37.7%, followed by second level teachers, unevaluated teachers, and senior teachers (28.5%, 16.6%, and 15.1%, respectively), while third level teachers account for the smallest part, only 2.1%. Moreover, 725 teachers are classroom teacher (46.5%) while 833 teachers are not (53.5).

### 3.2. Instruments for Data Collection

#### 3.2.1. Principals’ Instructional Leadership Scale

Our study adapted the Teaching Leadership Scale developed by Alig-Mielcarek and Hoy [52]. The scale consists of 3 dimensions, each of which has 1 or 2 questions. Dimension 1 is “support for teachers’ professional development” asking the degree to which principals encourage teachers to participate in professional training, and care about their teaching work and mental state. Dimension 2 is “data-driven teaching improvement”, asking the degree to which principals analyze student performance and plan student development goals together with teachers. Dimension 3 is “teaching feedback”, asking the frequency of principals’ feedback on teachers’ teaching. Each question uses a 6-point scoring system ranging from “strongly disagree” to “strongly agree”, with higher scores indicating higher levels of teaching leadership by the principal. The Cronbach’s Alpha coefficient is used to test the internal consistency reliability of scales in this study. It is the Cronbach’s alpha coefficient between 0.8 and 0.9 that indicates very good reliability. In this study, principals’ instructional leadership scale has good reliability, with a Cronbach’s alpha coefficient of 0.939.

#### 3.2.2. Teacher Role Stress Scale

Our study adapted the Role Stress Scale developed by Sharon Conley et al. [23], as well as the Role Conflict Scale developed by Richards [53]. The adapted scale consists of 9 questions divided into 3 dimensions: role ambiguity, role conflict, and role overload. Role ambiguity dimension focuses on teachers’ understanding about their role autonomy, responsibility and expectations from others. Role conflict dimension focus on the conflict between subject teaching and other work, and its effect on teachers. Role overload dimension focus on the level of busyness, time tension, and responsibility of teachers’ work. Each question uses a 6-point scoring system ranging from “strongly disagree” to “strongly agree”, with higher scores indicating greater levels of teacher role stress. In this study, the scale had good reliability, with a Cronbach’s alpha coefficient of 0.839.

#### 3.2.3. Teacher Affective Commitment Scale

Our study adapted the Affective Commitment Scale developed by Allen et al. [54]. The adapted scale consists of 7 single-dimensional structured items, concerning what extent are teachers willing to introduce and discuss their school with people; what extent teachers regard solving school difficulties as their responsibility; the significance of schools to teachers; teachers’ affective constraints, sustenance, and a sense of belongings towards their school. Each item is rated on a 6-point scale ranging from “completely inconsistent” to “completely consistent”, with higher scores indicating higher levels of teacher affective commitment. The reliability of the scale was good in this study, with a Cronbach’s alpha coefficient of 0.696.

#### 3.2.4. Teacher Retention Scale

Our study used a self-designed teacher retention questionnaire. The scale consists of 4 single-dimensional structured items: to what extent are teacher willing to stay at their current school, to leave education field, to regularly follow new work information, and to teach at a new school. Each item is rated on a 6-point scale ranging from “completely disagree” to “completely agree”, with higher scores indicating a stronger desire for teachers to stay. In our study, the second and fourth items were removed because of poor reliability, leaving only the first and third items. The reliability of the scale was good after this processing step was completed, with a Cronbach’s alpha coefficient of 0.651.

### 3.3. Data Processing

Our study used SPSS 25.0 and the PROCESS plugin as data analysis tools. The specific methods included reliability and validity testing, descriptive statistics, correlation analysis, and moderated chain mediation effect testing.

## 4. Results

### 4.1. Descriptive Statistics and Correlation Analysis

Each variable’s means, standard deviations, and correlation matrix are shown in Table 1. The correlation analysis indicated that there was a significant negative correlation (*p* < 0.01) between principals’ instructional leadership and role stress, between role stress and affective commitment, and between role stress and teacher retention; there was also a significant positive correlation (*p* < 0.01) between principals’ instructional leadership and affective commitment, between principals’ instructional leadership and teacher retention, and between affective commitment and teacher retention. These findings provided data support for further testing of the chain-mediation model.

### 4.2. Chain-Mediation MODEL Analysis

Based on the literature review and the relationships between variables, our study employed the SPSS macro program PROCESS plugin to test a chain-mediation model. In this model, principals’ instructional leadership is the independent variable, teacher retention is the dependent variable, and role stress and affective commitment are mediating variables. The Bootstrap method was used to draw 5000 samples from the sample (*n* = 1558), and the significance was judged based on whether the 95% confidence interval included 0. The results are shown in Table 2.

According to Table 2, principals’ instructional leadership significantly negatively predicts teacher retention (*β* = −0.102, *p* < 0.01) and significantly negatively predicts role stress (*β* = −0.245, *p* < 0.001); however, it significantly positively predicts affective commitment (*β* = 0.323, *p* < 0.001). Role stress significantly negatively predicts affective commitment (*β* = −0.269, *p* < 0.001) and teacher retention (*β* = −0.362, *p* < 0.001). Affective commitment significantly positively predicts teacher retention (*β* = 0.489, *p* < 0.001). The path coefficients for both separate and mediating effects are significant.

According to Table 3, the total effect of principals’ instructional leadership on teacher retention has a 95% confidence interval of [0.127, 0.226], which does not include 0. This means that principals’ instructional leadership significantly positively predicts teacher retention. The direct effect *c’* has a 95% confidence interval of [−0.151, −0.052], which also does not include 0, indicating a significant negative direct effect. The total indirect effect *ab* has a 95% confidence interval of [0.243, 0.315], which does not include 0, indicating a significant positive indirect effect. According to Wen, the indirect effect *ab* serves as a masking effect when the total effect *c*, the direct effect *c* and the indirect effect *ab* are significant, and the direct effect *c* and indirect effect *ab* are opposite in sign [55]. Therefore, in our study |ab|>|c’|, which means that it is true that principals’ instructional leadership directly negatively predicts teacher retention. However, it is the more significant mediating role of role stress and affective commitment that masks the direct effect, making the total effect significantly positive. In other words, the partially negative effect is only the remaining direct effect that is much weaker than the strong positive effects overall. Thus, principals’ instructional leadership ultimately significantly positively predicts teacher retention.

**Figure 2:**
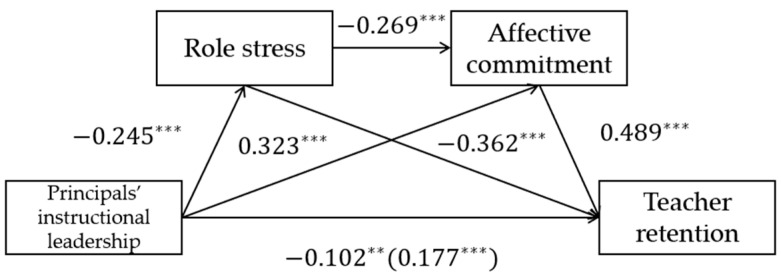
The Chain-Mediating Model. ** means *p* < 0.01; *** means *p* < 0.001.

Concretely, principals’ instructional leadership on teacher retention is influenced by three significant indirect paths. Path 1 has a confidence interval of [0.012, 0.112] and an effect size of 0.089, accounting for 87.3% of the total effect. Path 2 has a confidence interval of [0.004, 0.024] and an effect size of 0.032, accounting for 31.4% of the total effect. Path 3 has a confidence interval of [0.015, 0.129] and an effect size of 0.158, accounting for 154.9% of the total effect. Therefore, we confirm that principals’ instructional leadership affects teacher retention through the separate mediating role of role stress, the separate mediating role of affective commitment, and the chain-mediating role of role stress and affective commitment. The specific model is shown in Figure 2.

### 4.3. Moderated Chain Mediation Model Analysis

After Controlling variables of teaching experience and classroom teacher, we centralize measurement data. We use Model 92 from the SPSS PROCESS macro program and repeat sampling 5000 times, to test the moderating effect of gender in the chain mediation model. The results find that the interaction between teacher gender and principal teaching leadership has a significant negative impact on role stress (*β* = −0.133, *t* = −3.386, *p* < 0.001, *95%CI* = [−0.209, −0.056]), indicating that gender significantly negatively moderates the effect of principals’ instructional leadership on teacher role stress.

**Figure 3:**
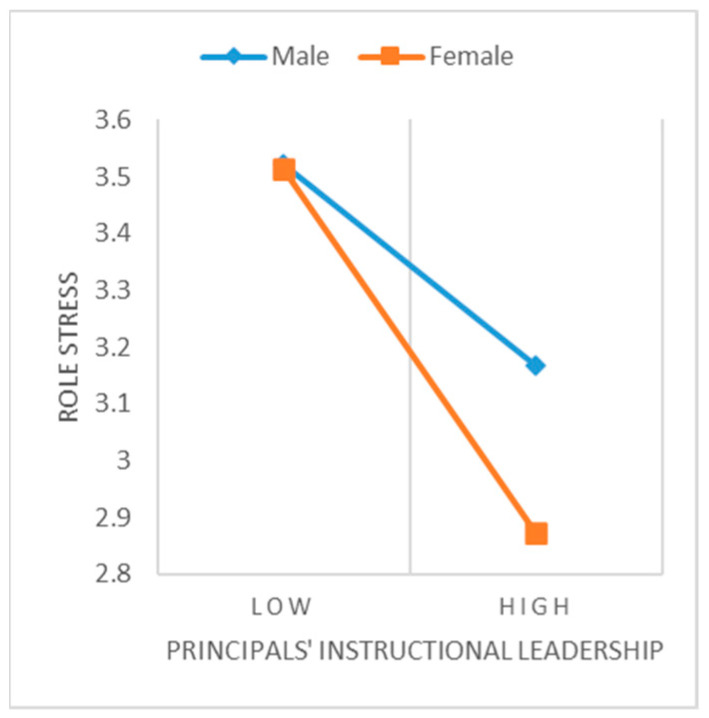
Moderating effect of gender.

Simple slope test method is used to further clarify the moderating effect of gender (shown in Figure 3). The result reflects that principals’ instructional leadership negatively predicts the role stress of male teachers, while this negative prediction is stronger for female teachers (*β* = −0.296, *t* = −11.843, *p* < 0.001, *95%CI* = [−0.345, −0.247]).

Further testing the moderated chain mediation model, as shown in Table 4, in the effect of principals’ instructional leadership on teacher retention through the chain-mediation role of role stress and affective commitment, there exists a gender difference. For male teachers, the chain-mediation effect value is 0.021, with a *95%CI* of [0.012, 0.033] excluding 0; while for female teachers, the chain-mediation effect value is 0.037, with a *95%CI* of [0.027, 0.050] excluding 0. Thus, the moderated chain mediating effect is significant, and Hypothesis 5 has been validated.

## 5. Discussion

This study aims to determine the relationship between principals’ instructional leadership and teacher retention. The results show that the direct and indirect effects of principals’ instructional leadership on teacher retention are significant. The indirect effects include three paths: principals’ instructional leadership affects teacher retention through the separate mediating role of role stress, the separate mediating role of affective commitment, and the chain-mediating role of role stress and affective commitment. The following discusses the results in detail.

### 5.1. The Situation of Principals’ Instructional Leadership, Role Stress, Affective Commitment, and Teacher Retention in Underdeveloped Regions of Central and Western China

Comparing the mean values of each factor with the theoretically neutral value reveals that in underdeveloped regions of Central and Western China, principals’ instructional leadership is at a relatively high level (Mean = 4.732), role stress is at a relatively low level (Mean = 3.245), and affective commitment (Mean = 4.291) and teacher retention (Mean = 4.860) are both at a relatively high level. These findings confirm that China’s policies have played a positive role in driving teacher retention in these regions [56]. However, only 37.1% of teachers in underdeveloped regions of Central and Western China fully agree with retaining their position at their current school for a long time, while only 52.4% of teachers believe that they are expected to remain in the education system after the next five years. These findings not only reflect the difficulty of recruiting, retaining and cultivating talent teachers in underdeveloped regions of Central and Western China [57] but also highlight that compared with leaving the education system altogether, these teachers are more likely to move from their current school to other positions within the education system [58].

### 5.2. Principals’ Instructional Leadership Partially Negatively PREDICTS Teacher Retention but Shows a Significant Positive Predictive Effect Overall

Our data fully support Hypothesis 1, and previous findings that principals’ instructional leadership significantly positively predicts teacher retention. However, there is inconsistency in that principals’ instructional leadership also partially negatively predicts teacher retention, although the negative effect is so weak that is ultimately suppressed by three paths containing two mediating variables.

On the premise of realizing that the effect of principals’ instructional leadership on teacher retention is mainly positive, that of the negative results, which are relatively weak, can be attributed to the special situation in these regions. Specifically, influenced by Confucian culture which emphasizes age, seniority, and social hierarchy, principals in these regions highlight principals’ roles as managers and politicians, rather than truly participating in teachers’ learning activities [7]. In times of rapid change, what promotes excellence today does not necessarily promote it or tomorrow [59]. The backwaters, rigidities, and conflicts in these regions may hinder transformational leadership. Thus, principals transform their leadership only when special problems related to tasks, performance, and organizational survival arise, rather than as a part of daily management. Then the partial negative effect occurs.

However, the positive predictive overall effect due to the suppressing effect of role press and affective commitment is more significant. Teacher retention can be seen as a behavioral manifestation of their role identity. In China, especially in these regions, the principal is at the top of the school organization “pyramid”, representing and shaping the school organization on their own, including regulations and cultures. Then the interaction between teachers and the principal can be regarded as the interaction between teachers and school structure, where teacher role identity is generated. Principals can exert instructional leadership by participating in teaching, perceiving teachers’ demands, and providing necessary emotional care. This type of instructional leadership may break the traditional hierarchical system between principals and teachers, influence traditional norms in schools, enhance teacher trust, and ultimately increase teacher retention.

### 5.3. Principals’ Instructional Leadership Affects Teacher Retention through the Mediating Role of Role Stress and Affective Commitment

Our data fully support Hypothesis 2, which states that role stress plays a mediating role between principals’ instructional leadership and teacher retention. Principals’ instructional leadership affects teachers’ understanding and perception of role stress through the school management system, which reflects structural role theory. In underdeveloped regions of Central and Western China, teachers are more prone to role stress due to the lack of security and humanistic care [60], and a heavy teaching burden is also an important obstacle to teacher retention [61]. Such work stress negatively affects teacher retention [24]. Furthermore, when young teachers lack the corresponding professional wisdom to perform their duties and cannot find a sense of purpose in their work, they will feel affectively exhausted [62]. The excessive expectations of teachers’ dedication are not conducive to the long-term development of excellent teachers in underdeveloped regions of Central and Western China [63]. Role conflict caused by teachers’ dual identity as both educators and parents inevitably leads to extended role conflict, which creates a sense of mission failure and affects teacher retention [26]. Exam-oriented education is regarded as an effective means of social mobility in underdeveloped regions of Central and Western China. Parents, students, principals, and society are extremely concerned about students’ academic performance. Based on the concept of “role sets” [64], this situation leads to teachers’ role stress. If principals can actively implement teaching reforms and reflect with teachers together, the level of teachers’ role stress can be effectively alleviated. Role identity theory also highlights that the degree of reward is the underlying exchange motivation for role identity, and role support is the most valuable reward. In underdeveloped regions of Central and Western China, teachers face role ambiguity and ecological confusion in classrooms [21]. As one of the sources of teachers’ role support, principals’ instructional leadership is related to the clarity of teachers’ teaching understanding, which affects their role identity and retention.

Our data fully support Hypothesis 3, which states that affective commitment plays a mediating role between principals’ instructional leadership and teacher retention. The results show that the expectation and support from principals can influence teachers’ affective attitude, effecting their identity to their own roles in school organizations. Focusing on these regions in China, previous studies have shown that the management system of schools in these areas is often more closed and rigid than that in other areas [65]. This management style can easily frustrate teachers’ autonomy, leading to negative emotions such as mistrust toward school and work. In the cultural context of China, leaders have a greater impact on subordinates than the organization itself, and loyalty to leaders is more important than loyalty to the system [12]. Effectively leading a complex and dynamic system requires principals to understand and respect how teachers work in their social and school contexts [66]. If principals in these regions can exert their instructional leadership and create an open organizational atmosphere, teachers are expected to develop more trust and loyalty toward the school, enhancing their affective commitment. Teachers with higher levels of affective commitment are also expected to have a sense of identification and belonging toward their school and be more eager to be retained [67].

Meanwhile, the demands–resource model suggests that individuals can convert resources from the situation into the psychological resources they possess [68]. In these regions, teachers obtain fewer resources for professional development. Studies have shown that in these areas, 74.1% of teachers report suffering from inadequate teaching materials and equipment, 70.8% of teachers report not having enough teaching reference materials, and 52.4% of teachers report not being able to obtain effective help when facing teaching difficulties [65]. Therefore, on the one hand, principals with high instructional leadership can provide more support for teachers’ professional development, give them encouragement and attention, enhance teachers’ psychological resources, and increase their affective commitment. On the other hand, teachers with high affective commitment can benefit from the resources provided by the school, and resource accumulation can further enhance their sense of belonging [45].

Our data fully support Hypothesis 4, which states that principals’ instructional leadership can influence teacher retention through the chain-mediating role of role stress and affective commitment. Role stress affects teachers’ role affect, role identification, and role well-being, which in turn influence their affective commitment, ultimately affecting teacher retention. Role affect drives the pursuit of an idealized self and can be constructed as teachers gain professional experience. In these regions, positive principals’ instructional leadership can alleviate role stress and enhance teachers’ affirmative emotions and constructive identification toward their role. During the new curriculum reform in China, teachers are facing an identity crisis, which manifests as deviation or ambiguity in direction, a lack of teaching individuality and creativity, and the demeaning of daily teaching. The essence of crisis is the lack of teachers’ subjective meaning [69], which reflects role ambiguity. Meanwhile, under the background of urbanization, teachers in underdeveloped regions of Central and Western China are no longer local intellectuals. Because urban teachers blindly follow in the process of professionalization, teachers’ creativity, judgment, and reflection face degradation risk in these areas [70]. Therefore, it is necessary for principals’ instructional leadership to alleviate teachers’ professional confusion or helplessness, cultivate their role awareness as educators, and ensure their affective commitment, ultimately enhancing their retention.

### 5.4. The Moderating Effect of Teacher Gender between Principals’ Instructional Leadership and Teacher Retention

Our data fully support Hypothesis 5, that teacher gender moderates the effect of principals’ instructional leadership on teacher role stress. It is true that this may be related to the gender imbalance in school teaching itself, that in our data, the proportion of female teachers is approximately twice that of male teachers. School teaching is often considered a female-dominated profession [71], which means not only the number of female teachers is often higher than that of men, but also teaching activities are related to typical female characteristics to some extent. However, rather than explaining that the gender imbalance affects the way teachers perceive and respond to stressors, it is better to regard gender as a representation of unique situations and a type of role conflicts in these regions. That is to say, it is situational factors such as local traditional culture, development level, organizational characteristics, and labor participation mode that are the deep reasons why female teachers have a more sensitive perception and response to principals’ instructional leadership. In these undeveloped regions in China, both the proportion of agricultural production personnel and that of labor force going out to work are relatively high, causing low labor productivity. Then time is a scarce resource in these regions [72], that many people have to overcome economic poverty by extending their working hours, but are trapped in time poverty. As to teachers, their multiple roles influenced by specific situation factors exacerbate role conflict and role overload, which is more common among female teachers. In local culture, traditional beliefs and role expectations emphasize the family responsibilities of females, even if they have their own profession, then the gender gap in time poverty is more serious [72]. Faced with the conflict of defining social identity as a family member or a social person, female teachers’ time is gendered and fragmented [51], so they have more difficulty than male teachers in coordinating the stress from multiple roles and potential role conflict caused [73]. If principals exert effective instructional leadership to provide teachers, especially female teachers, role support and identity, such as supporting them in lesson preparation, coordinate teaching work, and set some flexible system arrangements, female teachers’ role stress would be more significantly reduced. In contrast, the complex administrative tasks assigned by principals are expected to interrupt teacher’s teaching investment, seriously increasing their role stress, especially when combined with local situations.

## 6. Implications and Limitations

Our general conclusion is consistent with the existing research stating that principals’ instructional leadership positively predicts teacher retention. However, our study further validates the internal mechanism of principals’ instructional leadership to teacher retention on paper, emphasizing the critical role of this unique field. In underdeveloped regions of Central and Western China, social hierarchy is more emphasized, and principals often prioritize their political and managerial roles rather than truly engaging in teachers’ learning and communication [8], thus giving little attention to teachers’ role stress and affective commitment. Our study, however, demonstrates that the promotion of principals’ instructional leadership on teachers’ role stress and affective commitment may ultimately increase teacher retention in these areas, teacher gender also plays a moderating role in this process. This conclusion provides new perspectives and evidence for the identity construction of principals in underdeveloped regions of Central and Western China. However, our study has some limitations. Firstly, due to object limitations, our study did not collect multi-wave and multi-source data, which may lead to common method bias. What is more, our findings are limited by the sample size and have not been extended to schools, principals, or teachers in developed areas.

In terms of policy and practice, our findings suggest that more action is needed in underdeveloped regions of Central and Western China. First, schools in these areas urgently need to reform their system of principal appointment and selection, attach importance to the principal’s teaching and management abilities, and promote the benign participation of principals in instruction. Second, our study confirms that principals’ instructional leadership affects teacher retention through the mediating role of role stress and affective commitment. Therefore, principals in these areas need to ease teachers’ role overload, optimize their role cognition, and ensure their smooth role transitions at all stages. Principals also need to enhance teachers’ sense of belonging through humanistic nourishment and life care, abandon the mindset of relying on positional power, and provide more affective care to teachers. In summary, the Chinese government needs to cooperate with internal schools, especially the principals in these areas, to establish a more effective mechanism to promote the development of education in underdeveloped regions of Central and Western China.

## Figures and Tables

**Table 1:** Variable means, standard deviations, and correlation matrix.

Variable	M	SD	1	2	3	4
Principals’ instructional leadership	4.732	1.082				
Role stress	3.245	0.881	−0.284 **			
Affective commitment	4.291	0.863	0.506 **	−0.384 **		
Teacher retention	4.860	1.098	0.226 **	−0.409 **	0.488 **	

** means *p* < 0.01.

**Table 2:** Regression relationship between variables.

Result Variable	Predictive Variable	β	s.e.	t
Teacher retention	Principals’ instructional leadership	−0.102	0.025	−4.022 **
	Role stress	−0.362	0.030	−12.238 ***
	Affective commitment	0.489	0.033	14.838 ***
Role stress	Principals’ instructional leadership	−0.245	0.020	−12.429 ***
Affective commitment	Principals’ instructional leadership	0.323	0.018	18.253 ***
	Role stress	−0.269	0.022	−12.392 ***

** means *p* < 0.01; *** means *p* < 0.001.

**Table 3:** Chain-mediation analysis of role stress and affective commitment in principals’ instructional leadership and teacher retention.

	Effect Size	Boot ULCI	Boot LLCI	The Ratio of Indirect Effects to Direct Effects
Total effect	0.177	0.226	0.127	
Direct effect	−0.102	−0.052	−0.151	
Total indirect effects	0.279	0.315	0.243	273.5%
Path 1: Principals’ instructional leadership → Role stress → Teacher retention	0.089	0.112	0.012	87.3%
Path 2: Principals’ instructional leadership → Role stress → Affective commitment → Teacher retention	0.032	0.024	0.004	31.4%
Path 3: Principals’ instructional leadership → Affective commitment → Teacher retention	0.158	0.129	0.015	154.9%

**Table 4:** Chain mediating effects of different gender.

Moderator Variable	Indirect Effect	Boot SE	Boot LLCI	Boot ULCI
Male	0.021	0.005	0.012	0.033
Female	0.037	0.006	0.027	0.050

## Data Availability

The data presented in this study are available upon request from the corresponding author. The data are not publicly available due to confidentiality and research ethics.
